# Preparation and In Vitro Characterization of Magnetic CS/PVA/HA/pSPIONs Scaffolds for Magnetic Hyperthermia and Bone Regeneration

**DOI:** 10.3390/ijms24021128

**Published:** 2023-01-06

**Authors:** Francisco J. T. M. Tavares, Paula I. P. Soares, Jorge Carvalho Silva, João Paulo Borges

**Affiliations:** 1 i3N/CENIMAT, Department of Materials Science, NOVA School of Science and Technology (FCT NOVA), Campus de Caparica, 2829-516 Caparica, Portugal; 2 i3N/CENIMAT, Department of Physics, NOVA School of Science and Technology, Campus de Caparica, 2829-516 Caparica, Portugal

**Keywords:** 3D printing, additive manufacturing, bone regeneration, chitosan, magnetic hyperthermia, poly (vinyl alcohol), superparamagnetic iron oxide nanoparticles

## Abstract

Conventional bone cancer treatment often results in unwanted side effects, critical-sized bone defects, and inefficient cancer-cell targeting. Therefore, new approaches are necessary to better address bone cancer treatment and patient’s recovery. One solution may reside in the combination of bone regeneration scaffolds with magnetic hyperthermia. By incorporating pristine superparamagnetic iron oxide nanoparticles (pSPIONs) into additively manufactured scaffolds we created magnetic structures for magnetic hyperthermia and bone regeneration. For this, hydroxyapatite (HA) particles were integrated in a polymeric matrix composed of chitosan (CS) and poly (vinyl alcohol) (PVA). Once optimized, pSPIONs were added to the CS/PVA/HA paste at three different concentrations (1.92, 3.77, and 5.54 wt.%), and subsequently additively manufactured to form a scaffold. Results indicate that scaffolds containing 3.77 and 5.54 wt.% of pSPIONs, attained temperature increases of 6.6 and 7.5 °C in magnetic hyperthermia testing, respectively. In vitro studies using human osteosarcoma Saos-2 cells indicated that pSPIONs incorporation significantly stimulated cell adhesion, proliferation and alkaline phosphatase (ALP) expression when compared to CS/PVA/HA scaffolds. Thus, these results support that CS/PVA/HA/pSPIONs scaffolds with pSPIONs concentrations above or equal to 3.77 wt.% have the potential to be used for magnetic hyperthermia and bone regeneration.

## 1. Introduction

Osteosarcoma is a primary type of bone cancer, more frequently occurring in children and young adults, that can disrupt the osteoblastic and osteoclastic cell balance by stimulating focal bone deposition. Osteoblasts and osteoclasts are specialized cells responsible for bone formation and resorption, respectively, and the balance between them is at the base of bone remodeling. However, the disruption of these established dynamic processes leads to variations in bone mass, thus compromising bone renewal [1,2].

Managing patients with bone cancer, such as osteosarcoma, still poses a substantial clinical challenge that involves a multidisciplinary approach, which traditionally includes tumor resection, radiotherapy, and chemotherapy [3]. However, these procedures produce unwanted side effects and are not always sufficient to prevent tumor resurgence and the formation of non-osseous unions. Therefore, new strategies with the aim of improving a patient’s prognosis should include a successful bone defect reconstruction, the prevention of tumor resurgence, and the minimization of side effects [4,5,6].

Upon the diagnosis of bone cancer, patients that undergo tumor resection are often left with critical-sized bone defects, which will not heal spontaneously within the patient’s lifetime [7]. As a solution, a bone regeneration scaffold can be implanted, after the tumor’s removal, to support and enhance the body’s natural healing ability.

The use of additive manufacturing (AM), also termed 3D printing, in the production of these scaffolds, comes to address unmet clinical needs by allowing their customization through the control of the internal and external microarchitecture, thus ascribing unique properties and attaining better performances, through the improved mimicry or integration in heterogenous biological systems [8,9].

These scaffolds require materials with not only excellent mechanical properties to withstand various biomechanical forces, but must also be biocompatible, biofunctional, bioactive and biodegradable. Bioceramics are among the ideal materials for the fabrication of implants and scaffolds, owing to their similarity with bone mineral components, significant biocompatibility and osteointegration [10].

Hydroxyapatite (HA) represents 65–70% of bone’s mass and is, therefore, the main inorganic constituent of human bone [11]. Its application as a biomaterial has multiple advantages such as quick bone adaptation and intimate implant/tissue adhesion. However, its sole use as the scaffold’s matrix leads to a fragile structure prone to fracture. As a solution, HA particles can be incorporated into a polymeric matrix, which provides improved mechanical properties, while still benefiting from the HA’s bioactivity [12,13].

Polymers are ideal candidates for the fabrication of scaffolds due to the ease with which these can be shaped through the application of heat and/or pressure [13]. The combination of both natural (e.g., chitosan (CS)) and synthetic polymer (e.g., poly (vinyl alcohol), PVA) provides a structure with varying surface chemistry and improved properties such as mechanical strength, biocompatibility, and biodegradability [13,14,15].

CS is a semicrystalline polysaccharide commonly found in nature as the main derivative of chitin, present in crab and shrimp shells, and can be distinguished from chitin based on the amount of deacetylated units [16]. This natural polymer is biocompatible, abundant, and generates non-toxic degradation products during enzymatic hydrolysis. Furthermore, CS has suitable biological properties that facilitate osteoblastic cell adhesion and proliferation and can enhance the formation of a mineralized bone matrix [14,15,16,17,18]. However, pure CS hydrogel structures have low mechanical strength and ductility, making them unstable and prone to collapse. By combining CS with PVA, better performing mechanical features can be attained, deriving from intermolecular synergies [18,19].

PVA is a biocompatible synthetic polymer produced through the hydrolysis of poly (vinyl acetate), being the hydrolysis degree determined by the number of hydrolyzed units present. PVA exhibits low cytotoxicity, high chemical stability, good mechanical properties, and is easily dissolved in water. Moreover, PVA can be physically crosslinking by crystallization without toxic agents through freeze–thawing (F/T) cycles [18,20].

Although bone regeneration scaffolds composed of CS, PVA, and HA are excellent solutions for enhancing the body’s natural healing abilities, these do not prevent cancer resurgence. For this, a solution resides in the tumor’s characteristic low pO2 and pH environment, due to insufficient blood supply, which makes cancer cells highly susceptible to temperature, and its increase, in the range of 40–45 °C, promotes cell death by apoptosis [21,22].

To attain the necessary temperature gradient, magnetic nanoparticles (NPs) can be used due to their ability to generate heat by transducing an external alternating magnetic field (AMF) energy into thermal energy. This property can be used in vivo as a thermal treatment designated by magnetic hyperthermia (MHT) [23]. Furthermore, superparamagnetic NPs (e.g., pristine superparamagnetic iron oxide nanoparticles (pSPIONs)) exhibit fast change of their magnetic state with the application of an external AMF, presenting no remanence once the field is removed, at room temperature [8,24]. These characteristics make these NPs ideal for a new cancer approach that intends to personalize medicine for cancer patients [25,26].

The incorporation of magnetic NPs into an extrusion-based 3D printed scaffold allows for the creation of a system with MHT and bone regenerative capabilities, thus constituting a useful solution to overcome the side effects and critical-sized bone defects that derive from current treatments. Moreover, in localized bone cancer applications, such as osteosarcoma, better results can be attained by integrating pSPIONs into an artificial scaffold, thus creating magneto-responsive matrices that greatly enhance cell adhesion and osteogenic differentiation through magnetic stimulation [8].

Some authors, such as Zhang et al. [27] fabricated a magnetic composite scaffold composed of Fe_3_O_4_ NPs, mesoporous BG, and PCL, that made use of the BG mesoporosity to achieve excellent bioactivity and local drug delivery, while using the magnetite NPs for local MHT and osteoblast cell stimulation. Ergul et al. [19] studied the production of CS/PVA/HA scaffolds for bone regeneration using extrusion-based 3D printing. For this, four paste formulations with varying HA concentrations (2.5, 5, 10, and 15 wt.%) were tested for their printing quality. After printing and crosslinking the scaffolds, by spraying an NaOH/ethanol solution (7:3 ratio) onto them, the authors concluded that the 15 wt.% HA containing paste had the best performance. Following this, bone morphogenetic protein-2 (BMP-2) was added to the paste formulation and tested. The results showed clear adhesion and proliferation of human mesenchymal stem cells after a 72 h incubation period, but similar results were obtained for scaffolds with and without BMP-2 protein incorporation. Moreover, Liu et al. [18] successfully used extrusion-based 3D printing to produce CS/PVA scaffolds for drug delivery with adjustable drug release behavior based on the medium’s pH. To achieve this, CS and PVA were mixed at mass ratio of 3:5, 3:10, 3:15, and 3:30 combined with doxorubicin (DOX)-loaded carbon quantum dots and pre-crosslinked using genipin. Following printing, the scaffolds were submitted to F/T cycles for further crosslinking. The resulting system displayed a decrease of the swelling ratio and a clear increase of the hydrogel’s compressive strength with the increase of PVA content and proved that DOX release was higher for lower pH levels. However, few studies have been found that fully address the incorporation of HA and pSPIONs into a 3D printed scaffold with a CS/PVA matrix for applications in MHT and bone regeneration.

Therefore, in the present study we produced CS/PVA/HA scaffolds with incorporated pSPIONs, at three different concentrations, with the intent of evaluating their influence on the scaffolds’ properties and their potential for future application in MHT therapy. Furthermore, in vitro cytotoxicity, cell adhesion and proliferation, ALP expression and immunofluorescence studies were conducted to also evaluated the use of these scaffolds as bone regeneration scaffolds.

## 2. Results and Discussion

### 2.1. Paste Characterization

#### Paste Rheological Behavior

**Figure 1:**
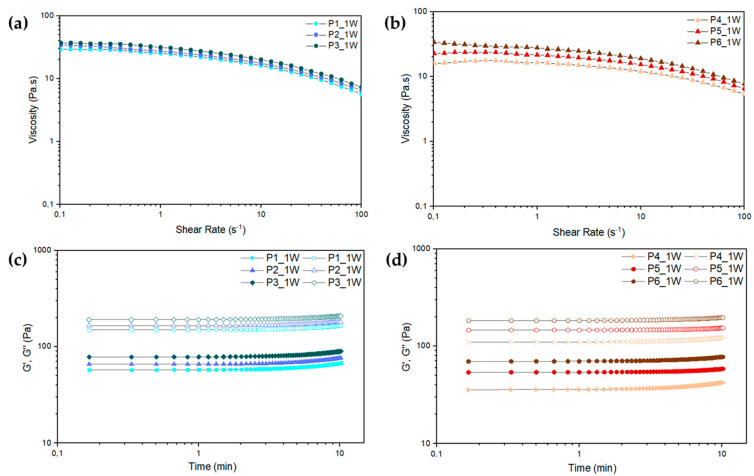
(**a**) Flow curves of pastes 1 to 3 after 1 week (W) of being produced; (**b**) Flow curves of pastes 4 to 6 after 1 week of being produced; (**c**) Oscillatory sweep curves of pastes 1 to 3 after 1 week; (**d**) Oscillatory sweep curves of pastes 4 to 6 after 1 week; Storage modulus (G′) represented by solid-colored forms and loss modulus (G″) represented by hollow-colored forms.

Rheological testing of the pastes was performed with the intent of studying the pastes’ viscosity at different shear rates, the elastic and viscous modulus (G′ and G″, respectively), and shelf life. For this, three replicas of each of the six different CS/PVA/HA formulations, presented in Table 1, were tested after production, thus obtaining the flow curves and oscillatory sweep curves presented in Figure 1. Since the time between paste production and 3D printing was of one week, these tests were conducted using that same timeframe, to better assess the pastes’ rheological properties once 3D printing started.

The flow curves, presented in Figure 1a,b, show a slight increase in viscosity with the increase of PVA concentration for all pastes, which can be explained by the increase in molecular chain interactions. Moreover, these pastes exhibit a shear-thinning behavior, thus facilitating the paste extrusion through a needle. This behavior occurs due to a reorganization of the polymer through a disentanglement of the polymeric chains. Besides this, shear-thinning pastes can act as suspension media for carrying cells or, for the purpose of this work, HA and pSPIONs [28,29].

Pastes were also submitted to dynamic testing to study if gelation would occur during the extrusion timeframe, which can seize extrusion through needle clogging. Oscillatory sweep curves, presented in Figure 1c,d, demonstrated that no gelation occurred within the tested timeframe of 10 min, which corresponds to double the timeframe of printing a 12-layer scaffold.

The paste systems’ stability was also studied through the course of eight weeks of being stored at room temperature. Throughout that same period, the rheological behavior of all tested pastes remained unaltered, hence their flow and oscillatory sweep curves remained the same as those presented for one week (see Figure 1a–d). The attained results indicate that the pastes are highly homogenous, thus evidencing a good CS and PVA blend and confirming that the sonication of HA influenced its stability in the paste, having not sedimented within the timeframe of the performed testing.

### 2.2. Paste Formulations Containing pSPIONs

Of all pastes tested, those with intermediate viscosities (P1 and P5) achieved better printability and filament shape retention. Moreover, paste P1 displayed the highest interlayer cohesion, due to the higher CS content in the paste, which allowed for a higher crosslinking density using the optimized NaOH/ethanol solution. As a result, paste P1 was chosen for further testing with the incorporation of pSPIONs.

pSPIONs were added to paste P1 at three different weight percentages relative to the total amount of HA (15, 30, and 45 wt.%) to produce pastes with the formulations presented in Table 2.

### 2.3. Scaffold Characterization

#### 2.3.1. Scanning Electron Microscopy (SEM)

**Figure 2:**
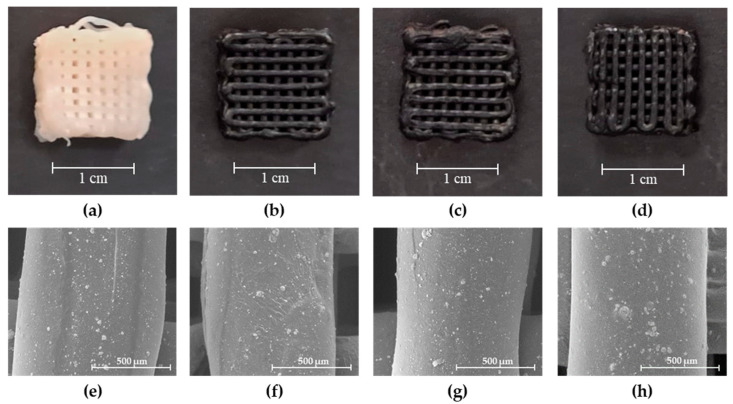
3D printed scaffolds after freeze-drying: (**a**) P1_B, (**b**) P1_pS1, (**c**) P1_pS3, and (**d**) P1_pS5; SEM images displaying the morphology and shape of the filaments obtained for the 3D printed scaffolds: (**e**) P1_B, (**f**) P1_pS1, (**g**) P1_pS3, and (**h**) P1_pS5. SEM images scale bar: 500 µm.

SEM imaging was performed to evaluate the surface morphology, scaffolds’ average filament diameter and shape fidelity. For this, the freeze-dried scaffolds, presented in Figure 2a–d, were coated with a chromium layer and observed through SEM.

As seen in Figure 2e–h, all scaffolds display defined structures with filament diameters similar to the 840 µm diameter of the needle used for their production (see Table 3). However, when comparing the average filament diameter of the control scaffold (P1_B) with those obtained for scaffolds containing pSPIONs (P1_pS1, P1_pS3, and P1_pS5), the latter three attained filament diameters that were closer to the inner diameter of the 18G needle used for 3D printing. Moreover, scaffolds with the highest concentration of pSPIONs (5.54 wt.%) attained minor diameter variability, whilst scaffolds produced without pSPIONs achieve structures with higher diameter variability.

This may be explained by the existing interactions between CS and the pSPIONs (as confirmed by ATR-FTIR data presented in Section 2.3.2) which helped improve the scaffolds’ shape retention of single filaments upon extrusion, as well as the resulting 3D structure when compared to the digital model.

On the surface of every produced scaffold, the presence of heterogeneously dispersed HA aggregates that added surface roughness to the polymeric matrix is noticeable. Furthermore, all scaffolds have dense filaments, which is linked to the post-printing treatment that involves the removal of excess glycerol (GLY) with ethanol, thus resulting in the dehydration of the structures prior to freeze-drying.

#### 2.3.2. Attenuated Total Reflectance—Fourier Transform Infrared Spectroscopy (ATR-FTIR)

ATR-FTIR spectroscopy was employed to characterize the scaffold components and attain information regarding the crosslinking between polymeric chains and the potential chemical bonding alterations derived from pSPIONs incorporation.

**Figure 3:**
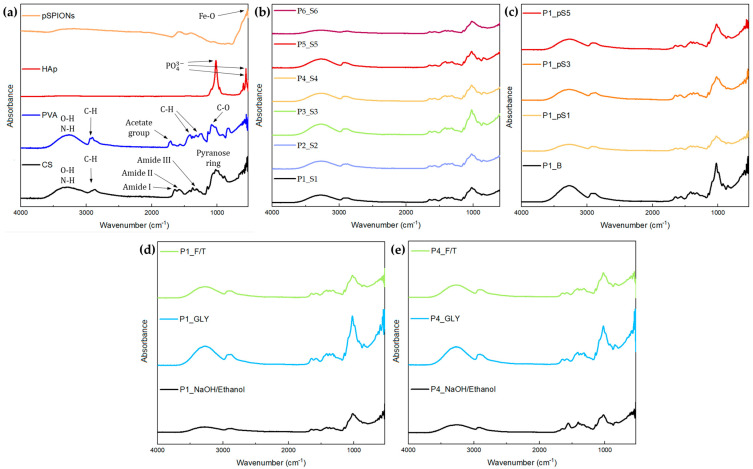
FTIR spectra: (**a**) pure CS (black), pure PVA (blue), pure HA (red), and pristine pSPIONs (orange); (**b**) P1_S1 (black), P1_S2 (blue), P1_S3 (green), P1_S4 (yellow), P1_S5 (red), P1_S6 (purple); (**c**) P1_B (black), P1_pS1 (yellow), P1_pS3 (orange), P1_pS5 (red). (**d**) FTIR spectra of P1_S1 at different moments in the post printing stage: Freeze dried scaffold after the NaOH/Ethanol solution (black); Freeze dried scaffold after the GLY bath and soaking in ethanol (blue); Freeze dried scaffold after undergoing 3 F/T cycles (green). (**e**) FTIR spectra of P4_S4 at different moments in the post printing stage: Freeze dried scaffold after the NaOH/Ethanol solution (black); Freeze dried scaffold after the GLY bath and soaking in ethanol (blue); Freeze dried scaffold after undergoing 3 F/T cycles (green).

CS spectrum, present in Figure 3a, displays a broad signal ranging between 3638 and 2991 cm^−1^ associated with O-H functional groups and N-H amino group stretching vibrations. A band observable at 2872 cm^−1^ derives from C-H stretching. Bands located at 1644, 1578, and 1317 cm^−1^ can be attributed to the presence of amide I, amide II, and amide III, respectively. Pyranose ring refers to saccharides with a ring like chemical structure composed of five carbons and one oxygen. It can be identified due to symmetric and asymmetric -${\mathrm{CH}}_{2}$- stretching at 2871, 1429, 1373, and 1251 cm^−1^ [17,19]. Moreover, major characteristic bands, observable at 1150, 1064, 1020, 983, and 892 cm^−1^, are ascribed to saccharide structures that compose the repeating unit of CS, having those bands at 1150 and 1064 cm^−1^ been also attributed to C-O stretching vibrations [30].

The PVA spectrum, observable in Figure 3a, exhibits a broad absorption band between 3600 and 2983 cm^−1^ that are also associated with OH functional groups and N-H amino groups stretching vibrations, and at 2909 cm^−1^ there is a band ascribed to saturated C-H stretching [17,30]. The band centred at 1711 cm^−1^ reveals C = O and C-O stretches, thus indicating the remanence of acetate groups despite the high hydrolysis percentage of 95%. This is followed by two bands at 1422 and 1332 cm^−1^ corresponding to C-H bending of -${\mathrm{CH}}_{2}$- and C-H deformation vibration, respectively. At 1081 cm^−1^ there is a large band related to the C-O stretching of acetyl group and at 827 cm^−1^ a band can be observed, which is attributed to C-C stretching vibrations [31].

Regarding the pure HA spectrum, three main characteristic bands can be observed in Figure 3a, at 1019, 603, and 558 cm^−1^, which can be attributed to ${\mathrm{PO}}_{4}^{3-}$ vibrations [32].

Figure 3d,e display the FTIR spectra for scaffolds P1_S1 and P4_S4, respectively, at three different stages of the post printing processes. CS and PVA blends might manifest a band between 1725 and 1700 cm^−1^ due to the production of carboxylic acid dimers which are originated by the acetic acid used to dissolve the CS [17,30]. This is more evident in the FTIR spectrum of P1_S1 than that of P4_S4 due to a higher CS concentration in P1_S1. Another feature of these blends is evidenced by a wider band between 3689 and 2976 cm^−1^ when compared to those verified for pure components, which results from the overlapping of the signals associated with O-H and N-H groups present in both polymers. Furthermore, the increase in PVA content, and subsequent increase in PVA crosslinking density through F/T cycles, promotes a shift of this band from 3290 (P1_S1) to 3261 cm^−1^ (P4_S4) [17]. This band shift is also slightly observable in Figure 3b, being that shift consistent with increase in PVA content, as previously mentioned. Besides this, the presence of HA is evident due to a high absorbance band at 1012 cm^−1^.

GLY acts as a plasticizer and promotes the formation of hydrogen bonds between CS and PVA [17]. GLY FTIR spectrum has characteristic bands at 3000 to 2800 cm^−1^ and 1500 to 1200 cm^−1^ regions associated with C-H bonds and a broad band between 3700 and 3000 cm^−1^ [30]. The presence of GLY is observable in both P1_S1 and P4_S4 spectra, presented Figure 3d,e, primarily due to an increase in the signal corresponding to hydroxyl groups, thus suggesting that the GLY bath achieved its purpose.

FTIR spectrum of pSPIONs, present in Figure 3a, displays a broad band between 3546 and 2900 cm^−1^ associated with the O-H stretching vibration, due to $H_{2}O$ vapor, and a band at 1604 cm^−1^ ascribed to O-H stretching vibrations. At 554 cm^−1^, a strong absorbance band is observable due to the Fe-O stretching vibration [33,34].

In Figure 3c, the characteristic band of pSPIONs, at 603, and 558 cm^−1^, is not perceptible due to their low concentration and the fact that Fe-O stretching vibration, characteristic of pSPIONs, falls under similar wavenumbers to those attributed to the ${\mathrm{PO}}_{4}^{3-}$ in HA. However, pSPIONs incorporation on the scaffolds leads to a slight shift in the band corresponding to the O-H and N-H groups, from 3264 (P1_B) to 3280 cm^−1^ (P1_pS5), due to electrostatic interaction between CS and pSPIONs [35]. Overall, when comparing Figure 3b,c, pSPIONs incorporation does not significantly change the chemical bonds of the scaffolds, despite a minor shift in the O-H and N-H group signal.

#### 2.3.3. Mechanical Characterization

To evaluate the scaffolds’ mechanical properties and study the influence of pSPIONs incorporation, the samples were submitted to compression tests. For this, samples P1_B and P1_pS5 with four layers each were replicated five times. Before being submitted to compression testing using a 100 N load cell at 2 mm/min, the samples were prepared by cutting them into 1 cm^2^ squares and soaking these in a PBS solution overnight.

**Figure 4:**
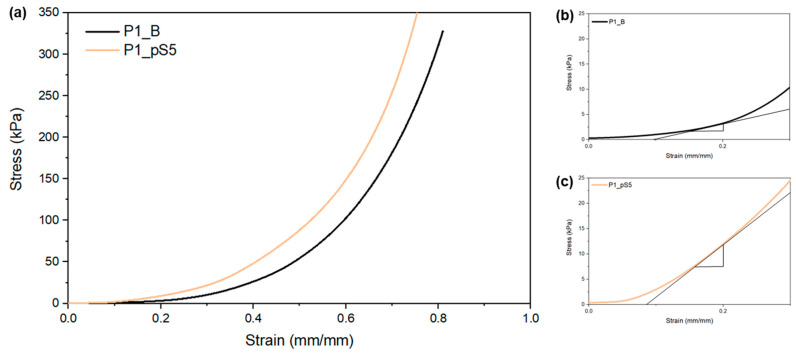
(**a**) Compression stress–strain curves for scaffolds with 5.54 wt.% of pSPIONs (P1_pS5 (orange)) and without pSPIONs (P1_B (black)); (**b**) Enlargement of the compression stress-train curve obtained for scaffolds P1_B, evidencing the slope at 20% strain; (**c**) Enlargement of the compression stress-train curve obtained for scaffolds P1_pS5, evidencing the slope at 20% strain.

The compression stress–strain curves obtained for all tested scaffolds (see Figure 4a) indicate an increase in mechanical strength with the increase of strain, which is in agreement with studies carried out by Liu et al. [18] and Ergul et al. [19], who investigated scaffolds of similar matrix compositions to those in the present study.

These types of compression stress–strain curves, designated as J-shaped curves, are characterized by their exponential behavior and are typical of biological soft tissues mainly composed of type I collagen, such as skin and the organic phase of bones [36]. Under normal physiological conditions, soft tissues are usually exposed to mechanical efforts comprised within the toe region [37].

Figure 4b,c, which consist of an enlargement of the compression stress–strain curves presented in Figure 4a, display the difference between their toe modulus (attained as the slope at 20% strain). By comparing the toe modulus presented in Table 4, a significant variation is observable when comparing scaffolds with and without pSPIONs incorporation. This could be attributed to the electrostatic interactions between CS and pSPIONs, which have a significant impact on the mechanical properties of the scaffold, thus confirming that the pSPIONs act as a reinforcement.

#### 2.3.4. Magnetic Hyperthermia (MHT)

**Figure 5:**
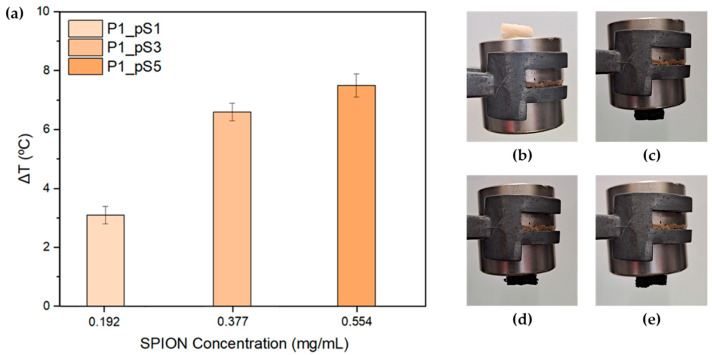
(**a**) Total temperature increase of the environment surrounding the scaffolds after 10 min for scaffolds containing 1.92, 3.77, and 5.54 wt.% of pSPIONs. Preliminary magnetic property testing under a magnet for a: (**b**) scaffold without pSPIONs (P1_B), (**c**) scaffold with 1.92 wt.% of pSPIONs (P1_pS1), (**d**) scaffold with 3.77 wt.% of pSPIONs (P1_pS3), and (**e**) scaffold with 5.54 wt.% of pSPIONs (P1_pS5).

Prior to MHT testing, all printed scaffolds were tested their response under a magnet. As observable in Figure 5b–e, all pSPIONs containing scaffolds displayed magnetic properties under a magnetic field, while none were observed for scaffolds composed of only base material (CS, PVA, and HA).

MHT characterization of the scaffolds was performed with the intent of evaluating the pSPIONs’ heating capacity when incorporated into a CS/PVA/HA scaffold (P1_B). For this, three replicas of each pSPIONs containing scaffold were prepared by adding 10 mg to 1 mL of MilliQ water and left to swell overnight. As mentioned before, these results were obtained by submitting the scaffolds to a magnetic flux density of 300 Gauss and a frequency of 418.5 kHz, for 10 min.

Figure 5a shows the total temperature increase of the environment surrounding the pSPIONs containing scaffolds at theoretical concentrations of 1.92, 3.77, and 5.54 wt.%. Scaffolds containing pSPIONs displayed an increase in temperature with the increase of their concentration. When comparing the scaffolds with different concentrations of pSPIONs, it is noticeable that scaffolds with pSPIONs concentration of 3.77 wt.% and above display higher heating capacities, having reached temperature increase of 6.6 and 7.5 °C, for concentration of 3.77 and 5.54 wt.%, respectively. Finally, the structures with a pSPIONs concentration of 1.92 wt.% registered the lowest temperature increase.

These results can be explained by the amount of pSPIONs present in each scaffold. This relation states that an increase in pSPIONs quantity leads to an increase of the temperature of the surrounding medium where the scaffold is present and submitted to an AMF [8,34]. Therefore, scaffold P1_pS5 which contains 5.54 wt.% of pSPIONs exhibits a higher temperature increase compared to the other scaffolds with a smaller amount of pSPIONs.

Regarding MHT applications for cancer therapy, the structures should be able to increase the local body temperature to a range of 40–45 °C. To guarantee that the scaffolds reach the right temperatures, it is assumed that they should reach a minimum of 42 °C to be deemed usable for MHT. Moreover, assuming that the average body temperature is 37 °C, the scaffolds should be able to achieve a minimum temperature increase of 5 °C. Following these criteria, the scaffolds with 1.92 wt.% of pSPIONs, which reached a 3.1 °C increase, were the only scaffolds that could not reach the 5 °C minimum.

Despite this, it is important to state that the current analysis was conducted regarding the results obtained for 10 mg scaffolds. Therefore, should the sample size be increased, a higher temperature increase of the environment surrounding the scaffold would be expected.

#### 2.3.5. Swelling Ratio and Scaffold Erosion

**Figure 6:**
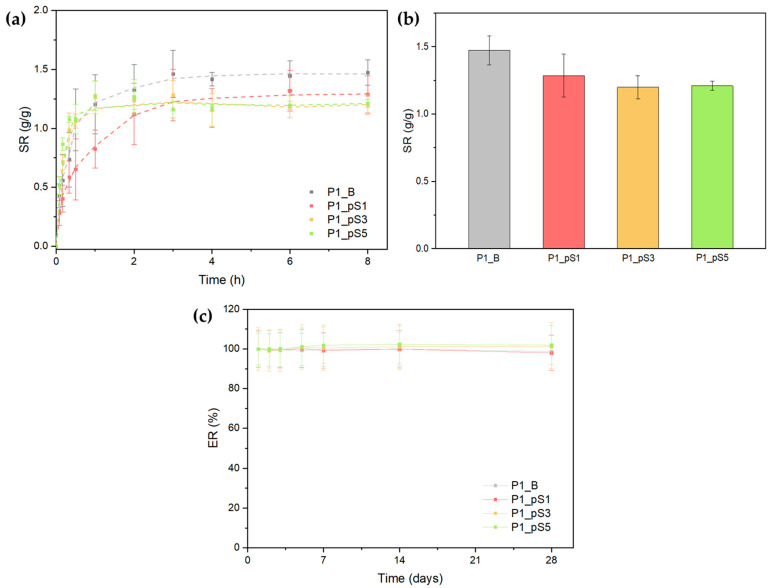
(**a**) Swelling ratio curves for scaffolds without pSPIONs (P1_B (grey)), scaffolds containing 1.92 wt.% of pSPIONs (P1_pS1 (magenta)), scaffolds containing 3.77 wt.% of pSPIONs (P1_pS3 (yellow)), and scaffolds containing 5.54 wt.% of pSPIONs (P1_pS5 (light green)) samples; (**b**) Scaffolds’ swelling ratio after 8h, following the same color scheme; (**c**) Erosion curves for P1_B, P1_pS1, P1_pS3, and P1_pS5 samples, following the same color scheme.

Figure 6a displays the swelling curves of scaffolds containing pSPIONs and P1_B as a control. All curves exhibit a logarithmic behavior, and the swelling ratios tends to stabilize after 3 h in a PBS solution. Through the observation of Figure 6b, which highlights the swelling ratio after 8 h in a PBS solution, it is possible to affirm that the highest swelling ratio was registered for the P1_B samples, at 1.47 (g/g).

Overall, there seems to be a swelling ratio decrease with the increase in pSPIONs concentration. This could derive from the fact that the presence of pSPIONs in the scaffolds promotes electrostatic interactions between pSPIONs and CS, as confirmed by ATR-FTIR data. In turn, these interactions restrain the structures’ ability to attract water towards itself, thus resulting in a lower swelling ratio. However, ATR-FTIR data also confirms that these electrostatic interactions are limited by the number of pre-existing CS-GLY interactions, which may explain the similarity between the swelling ratios obtained for scaffolds P1_pS3 and P1_pS5, with 1.20 and 1.21 (g/g), respectively.

As observable in Figure 6c, all scaffolds display negligible mass variations, therefore it is possible to affirm that close to no erosion occurred within the tested timeframe. These results further confirm that the scaffolds crosslinking, achieved by a combination of NaOH (CS), GLY (between CS and PVA), and F/T cycles (PVA), was successful in improving their stability, by guaranteeing a negligible amount of polymeric and pSPIONs leaching from the structures. Moreover, the incorporation of pSPIONs did not have a significant impact on the scaffolds’ structural integrity, as expected.

### 2.4. In Vitro Studies

#### 2.4.1. Cell Viability Assays

As reported in the literature, the use of pSPIONs comes with issues regarding their stabilization, which is crucial against aggregation in a biological medium and a magnetic field. Besides this, freely dispersed iron oxide NPs in culture medium are not cytotoxic below concentrations of 1.0 mg/mL [8,34]. However, in vitro testing was conducted using initial pSPIONs concentrations of 1.92, 3.77, and 5.54 mg/mL for scaffolds P1_pS1, P1_pS3, and P1_pS5, respectively.

Knowing this, cell viability assays were conducted to evaluate the potential cytotoxicity effect of the scaffolds containing pSPIONs using the human osteosarcoma Saos-2 cell line and the extract method with serial dilutions. Furthermore, since the biocompatibility of the base materials (CS, PVA, and HA) has been extensively studied, any cytotoxicity observed may derive from reaction by-products, pSPIONs concentration or residue from post-printing processes.

**Figure 7:**
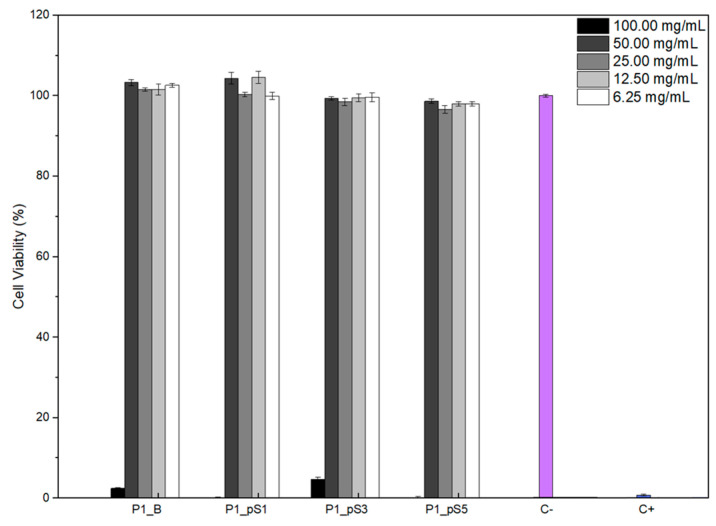
Cell viability studied using resazurin assays to assess the structures’ cytotoxicity effect on a Saos-2 (primary osteogenic sarcoma) cell line using the extract dilution exposure method, after 48 h of cell exposure to the scaffolds’ extracts: scaffolds without pSPIONs (P1_B), scaffolds containing 1.92 wt.% of pSPIONs (P1_pS1), scaffolds containing 3.77 wt.% of pSPIONs (P1_pS3), and scaffolds containing 5.54 wt.% of pSPIONs (P1_pS5); A positive control (C+) using dimethyl sulfoxide (blue) and negative control (C−) (purple).

Results presented in Figure 7 display the cell viability for each scaffold composition and the five different extract concentrations tested. These indicate that all scaffolds are cytotoxic at 100 mg/mL. However, at a concentration of 50 mg/mL, achieved after the first dilution, every scaffold displayed a cell viability above 90%, thus indicating no cytotoxicity. These results indicate that the incorporation of pSPIONs does not decrease cell viability and that the cytotoxicity verified at 100 mg/mL should, therefore, result from reaction by-products or residue from post-printing processes. Moreover, the lack of cytotoxic effect derived from the presence of pSPIONs in the scaffolds at concentrations above 1.0 mg/mL, corroborates erosion testing data suggesting that the release of pSPIONs was negligible.

As a solution to the cytotoxicity exhibited at 100 mg/mL, scaffolds can be soaked in a PBS solution for 24 h, as suggested by Silva et al. [38].

#### 2.4.2. Cell Adhesion and Proliferation

**Figure 8:**
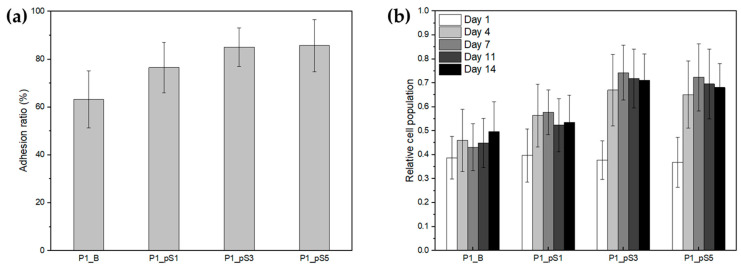
(**a**) Comparison of the human osteosarcoma Saos-2 cells adhesion ratios to the four produced scaffolds: without pSPIONs (P1_B), containing 1.92 wt.% of pSPIONs (P1_pS1), containing 3.77 wt.% of pSPIONs (P1_pS3), and containing 5.54 wt.% of pSPIONs (P1_pS5); (**b**) Comparison of cellular proliferation of human osteosarcoma Saos-2 cells cultured for 14 days on three different CS/PVA/HA/pSPIONs scaffolds and on CS/PVA/HA scaffolds as control. Absorbance values are normalized to the average absorbance of the control wells on day 1. The vertical lines represent the experimental standard deviation of the mean.

The adhesion and proliferation of human osteosarcoma Saos-2 cells were determined for scaffolds P1_B, P1_pS1, P1_pS3, and P1_pS5 to infer their ability to assist tissue regeneration in vivo. The adhesion ratios calculated using Equation (7) are shown in Figure 8 and presented in Table 5.

Scaffolds P1_pS3 and P1_pS5 attained the highest adhesion ratios of (85 ± 8)% and (86 ± 11)%, respectively, followed by scaffolds P1_pS1 with (77 ± 11)% and P1_B scaffolds registering the lowest adhesion ratio of (63 ± 12)%. These results indicate that there is an increase in cell adhesion with the increase in pSPIONs content, being that increase more statistically significant for scaffolds with the highest pSPIONs content, P1_pS3 and P1_pS5, in relation to the control (P1_B).

These results were expected since PVA is known to have low protein affinities, which results in poor cell-surface interaction [39,40]. However, the incorporation of pSPIONs significantly promoted cell adhesion and proliferation as observable in Figure 8a,b. This can be attributed to the fact that pSPIONs may be more conducive to the adsorption of vitronectin, an abundant glycoprotein crucial for bone cell adhesion, thus facilitating subsequent pSPIONs and cell membrane interactions by triggering key integrin receptors [41,42].

Figure 8b displays the relative cell population growth from day 1 to day 14, having the data collected for days 11 and 14 possibly been influenced by the well change performed on days 7 and 11, which may have stressed the cells on the scaffolds, thus resulting in a stagnation of their proliferation. 

Table 6 shows the cell populations on the three pSPIONs-containing scaffolds, material control, and cell control for days 1, 4 and 7, as well as the proliferation ratios: PR 1-4, ratio between cell population on days 4 and 1; PR 1-7, calculated similarly between days 7 and 1.

From day 1 to 4, cell populations increased on all scaffolds, thus suggesting that all of the tested formulations support cell proliferation. Cell growth on scaffolds P1_pS3 and P1_pS5, exhibited the highest population increase (between 80%–100%) which is similar to that of the cell control. Those grown on scaffolds P1_pS1 had an increase around 51%, having the cells grown on scaffolds P1_B obtained the lowest population increase, of around 21%.

When comparing the population increases obtained for a larger timeframe, day 1 to day 7, this difference becomes more evident, with scaffolds P1_pS3 and P1_pS5 registering a population increase between 85%–110%, and scaffolds P1_B and P1_pS1 having a population increase between 10%–25%. This may also derive from the low affinity of the PVA towards proteins, which limits the ability for new cells to adhere to the scaffold, thus hindering cell proliferation. Furthermore, this may also confirm that the integration of pSPIONs significantly improves cell adhesion and increases cell proliferation.

#### 2.4.3. Relative ALP Expression of Saos-2 Cells on CS/PVA/HA/pSPIONs Scaffolds

Alkaline phosphatase, an enzyme which acts as a catalyst, is known to regulate the local concentration of inorganic phosphate and is able to catalyse the growth of apatite crystals, essential for biomineralization [43,44,45].

**Figure 9:**
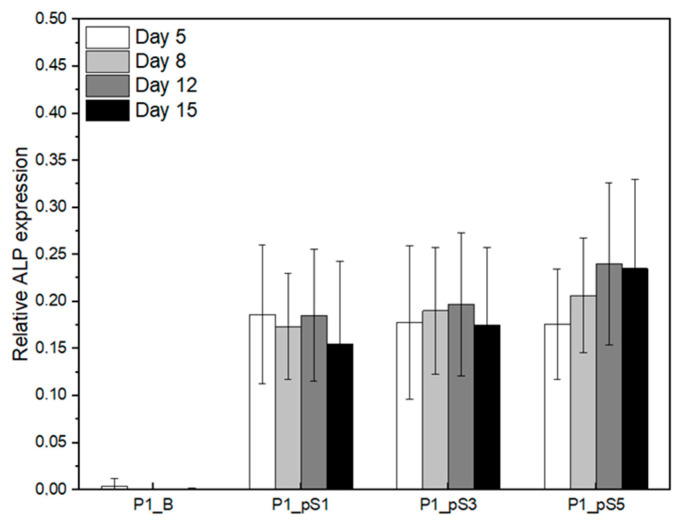
Relative ALP expression of Saos-2 cells on scaffolds: without pSPIONs (P1_B), containing 1.92 wt.% of pSPIONs (P1_pS1), containing 3.77 wt.% of pSPIONs (P1_pS3), and containing 5.54 wt.% of pSPIONs (P1_pS5).

Figure 9 displays the relative ALP expression of the Saos-2 cells grown on the four different scaffolds throughout the 15 days of culture time. All cells grown on scaffolds revealed relative ALP expression with the exception of those grown on the material control scaffolds (P1_B), for which the ALP level measured in the culture medium was similar to that obtained for fresh McCoy’s 5a medium supplemented with fetal bovine serum. This basal level of ALP in the culture medium was subtracted from the values measured. Therefore, any ALP expression by cells grown on P1_B scaffolds was residual. On the contrary, cells grown on scaffolds containing pSPIONs had detectable ALP expression, being that expression statistically significant when comparing with the material control, as presented in Table 7. Furthermore, this indicates that the cells grown on scaffolds P1_pS1, P1_pS3, and P1_pS5 will probably contribute to the production of the inorganic phase of bones through the formation of an apatite layer. However, the ALP activity of the cells grown on the pSPIONs-containing scaffolds did not significantly vary with the increase in pSPIONs concentration nor with time.

#### 2.4.4. Cell Morphology

**Figure 10:**
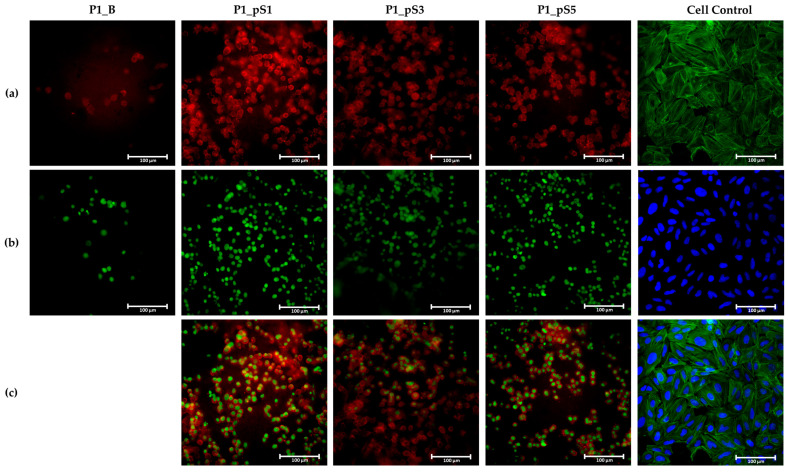
Fluorescence of Saos-2 cells on scaffolds without pSPIONs (P1_B), containing 1.92 wt.% of pSPIONs (P1_pS1), containing 3.77 wt.% of pSPIONs (P1_pS3), and containing 5.54 wt.% of pSPIONs (P1_pS5) using Phalloidin CruzFluor™ 488 Conjugate, Helix NP Green and DAPI where: (**a**) Cytoskeleton of the cells on top of the scaffolds (red) and cytoskeleton of the cell control (green); (**b**) Nucleus of the cell on top of the scaffolds (green) and nucleus of the cell control (blue); and (**c**) Merge of the images obtained for the cytoskeleton and nucleus. Scale size: 100 µm.

Fluorescence microscopy images taken after 15 days of culture, shown in Figure 10, confirm the presence of attached cells to the surface of all tested scaffolds. Cell control images, presented in Figure 10, were taken after 1 day of culture time to better demonstrate the Saos-2 cell line morphology without the influence of high cell confluency. Furthermore, the polymers that compose the scaffolds’ matrix (CS and PVA) displayed autofluorescence when observed using the red channel. However, that autofluorescence was attenuated by the presence of pSPIONs, which significantly improved the quality of the obtained imagens Figure 10a.

Regarding cell morphology, the cell control after 1 day of culture displays the typical Saos-2 cell line morphology which can be described as polygonal or epithelial-like geometry [46,47]. Moreover, these occupy a large amount of the available surface area which indicates an excellent interaction with the substrate and consequently allows for a better organelles’ organization [48]. Contrastingly, cells grown on the material control scaffolds (P1_B) display a rounded geometry, a smaller nucleus, and occupy a surface area that is only slightly larger than that of its nucleus, as evidenced in Figure 10a,c. This can be attributed to PVA which is known for its low protein affinity, thus resulting in poor cell-surface interaction by limiting the binding of the secreted extracellular matrix that supports cell attachment [39,40]. 

Despite having shown better adhesion ratios and proliferation rates than those attained by scaffolds P1_B, cells grown on scaffolds containing pSPIONs display a morphology similar to that of the cell grown on the material control scaffolds. This was expected since the PVA content does not significantly change between scaffolds with pSPIONs incorporation, which means that the PVA within the structures still significantly influences the physicochemical properties of the scaffolds’ surface and its’ protein affinity.

## 3. Materials and Methods

### 3.1. Materials

Iron (II) chloride tetrahydrate (${\mathrm{FeCl}}_{2}\;.\;4H_{2}O$, 98%) was purchased from Alfa Aesar (Ward Hill, MA, USA). Iron (III) chloride hexahydrate (${\mathrm{FeCl}}_{3}\;.\;6H_{2}O$, 97%) and hydroxyapatite (${\mathrm{Ca}}_{10}{\left({\mathrm{PO}}_{2}\right)}_{6}{\left(\mathrm{OH}\right)}_{2}$) were purchased from Sigma-Aldrich (St. Louis, MO, USA). Ammonium hydroxide solution (${\mathrm{NH}}_{4}\mathrm{OH}$, 25%) was purchased from Honeywell Fluka^TM^ (Charlotte, NC, USA). Glacial acetic acid (${\mathrm{CH}}_{3}\mathrm{COOH}$) was purchased from Fisher Chemical (Waltham, MA, USA). Chitosan (${\left(C_{8}H_{13}{\mathrm{NO}}_{5}\right)}_{n}$, $M_{w}=50,000\;\mathrm{to}\;1,000,000$, 70 % deacetylation) was purchased from Cognis (Monheim am Rhein, Germany). Poly (vinyl) alcohol (${\left(C_{2}H_{4}O\right)}_{n}$, $M_{w}=95,000$, 95% hydrolyzed) was purchased from Acros Organics (Geel, Belgium). Sodium hydroxide was purchased from Eka (Eka Pellets^TM^, Nouryon, Amsterdam, The Netherlands). Ethanol 96% was purchased from LabChem (Zelienople, PA, USA).

Regarding the materials used for cell culture; Resazurin was purchased from Alfa Aesar (Ward Hill, MA, USA). McCoy 5A medium and fetal bovine serum were purchased from BioWest (Nuaillé, France). Triton X-100 and 4-nitrophenyl phosphate disodium salt hexahydrate (pNPP) were purchased from Sigma-Aldrich (St. Louis, MO, USA). Saos-2 cells were purchased from ATCC (Manassas, VA, USA). Penicillin-Streptomycin (10,000 mL^−1^) was purchased from Gibco (ThermoFisher Scientific, Waltham, MA, USA). Paraformaldehyde (PFA) was purchased from Scharlab (Barcelona, Spain). DAPI was purchased from Invitrogen (ThermoFisher Scientific, Waltham, MA, USA). Phalloidin CruzFluor^TM^ 488 Conjugate was purchased from Santa Cruz Biotechnology (Dallas, TX, USA). Helix NP^TM^ Green was purchased from Biolegend (San Diego, CA, USA).

### 3.2. Methods

#### 3.2.1. Pristine Superparamagnetic Iron Oxide Nanoparticles (pSPIONs) Synthesis

Pristine superparamagnetic iron oxide nanoparticles (pSPIONs) were synthesized by chemical co-precipitation using ferrous and ferric chlorides and following the method described by Soares et al. [33]. Following the production of the pSPIONs, these underwent dialysis (MW 12–14 kDa, Spectrum^TM^ Spectra/Por^TM^), to remove unreacted reagents and attain a neutral pH.

#### 3.2.2. Paste Preparation

A 6 (%*w*/*V*) CS paste was produced by dissolving CS in a 1.5 (%*V*/*V*) glacial acetic acid solution, using a mechanical stirrer for 90 min. To produce the PVA/HA pastes, hydroxyapatite was added to MilliQ water, at 15 wt.% of the total amount of polymer in the paste and submitted to ultrasounds (Hielscher ultrasonic processor UP400St, Teltow, Germany) at 100% cycle for 2 min. Following this, PVA was added in three different weight-to-volume percentages (12.5, 15, and 17.5 %*w*/*V*) to the suspension and left, under mechanical stirring at 85 °C, for 90 min. Then, the CS and PVA/HA pastes were combined to produce six different pastes, three with volume ratio of 3:1 and other three of 3:2. To produce the pastes containing pSPIONs, these were added to the CS paste prior to mixing with the PVA/HA paste, at three different weight percentages (15, 30, and 45 wt.%) relative to the total amount of HA in the paste.

#### 3.2.3. Three-Dimensional (3D) Printing and Post-Printing Treatments

The production of the scaffolds was carried out with a Zmorph VX 3D (Zmorph, Wroclaw, Poland) printer modified by the authors. SolidWorks 2020 (Waltham, MA, USA) was used to create a wave pattern, which was then sliced using the printer’s recommended slicing software (Voxelizer 2.0, Wroclaw, Poland). Finally, the 3D model’s G-code was written to create a 12-layer scaffold with alternating wave layers rotated 90° in relation to one another. 

The hydrogel was loaded into a 10 mL syringe with an 18G needle and set on the 3D printer. While printing the paste onto a Petri dish, a solution composed of NaOH at 0.4 M and 96% grade ethanol, combined using a ratio of 7:3, was manually dripped onto the scaffold [19,49]. Once all 12 layers were printed, more NaOH/ethanol solution was added to fully submerge the structure for 5 min. All prints were performed at room temperature and at a print speed of 10.5 mm/s.

Following the NaOH/ethanol bath, the scaffolds were dried using paper before being submerged in a glycerol (GLY) bath for 30 min. Once removed from the bath, the excess GLY was patted from the structure with paper. To avoid the presence of excess GLY in the structure, the scaffolds were submerged in ethanol (96%) overnight. After ethanol evaporation from the structures, the scaffolds were submitted to 3 F/T cycles of 12 h each at −20 °C. Then, samples were left in the freezer overnight to be freeze-dried (VaCo 2, ZIRBUS, Bad Grund, Germany) at −43 °C and a pressure of 0.11 mbar for 48 h.

#### 3.2.4. Paste Characterization

##### Rheological Behavior

Rheological experiments of the composite pastes were carried out at 25 °C using an Anton Paar MCR 502 rheometer (Anton Paar, Graz, Austria) with parallel plate geometry (PP25) and a 1 mm gap. The rheological analyses were performed in steady shear mode at shear rates ranging from 0.1 to 100 s^−1^ and dynamic testing was conducted using a frequency of 10 rad/s for 600 s. For this, 3 replicas of each of the six different previously mentioned CS/PVA/HA formulations were tested 1, 4, and 8 weeks (W) after production, thus obtaining their characteristic flow curves and oscillatory sweep curves. 

#### 3.2.5. Physicochemical, Morphological, and Magnetic Characterization of the Scaffolds

##### Scanning Electron Microscopy (SEM)

The studied scaffolds were sputter coated with a thin layer of chromium for the analysis of their filament diameter and surface morphology, employing a tabletop microscope TM3030 Plus Hitachi (Hitachi, Tokyo, Japan). In order to obtain the average filament diameter for each of the four different scaffold compositions, as shown in Table 3, the diameter of 15 filaments belonging to different scaffolds was measured using ImageJ software [50].

##### Attenuated Total Reflectance—Fourier Transform Infrared Spectroscopy (ATR-FTIR)

FTIR spectra were obtained using a Thermo Nicolet 6700 FTIR spectrometer (Thermo Scientific, Waltham, MA, USA) in the wavenumber range of 4000–525 cm^−1^ with a resolution of 2 cm^−1^. The use of ATR allowed for a direct measurement of the samples in the solid state without previous preparation.

##### Magnetic Hyperthermia (MHT)

MHT measurements of the pSPIONs containing scaffolds were performed using a nanoScale Biomagnetics, DM100 Series (nanoScale Biomagnetics, Zaragoza, Spain) with a magnetic flux density of 300 gauss and a frequency of 418.5 kHz, for 10 min. For this, three replicas of each scaffold composition (containing 1.92, 3.77, and 5.54 wt.% of pSPIONs) were previously prepared through the addition of 10 mg of scaffold to 1 mL of MilliQ water and left to swell overnight.

##### Mechanical Characterization

Mechanical characterization was performed to assess the influence of pSPIONs on the scaffolds’ mechanical properties under compressive forces. For this, samples of 4-layered scaffolds were prepared by cutting them into 1 cm^2^ squares and soaking these in a PBS solution overnight. Then, a static-dynamic testing machine (Inspekt micro-LC 100N, Hegewald & Peschke, Nossen, Germany) was used to test 5 replicas per scaffold composition using a 100 N load cell at a compression rate of 2 mm/min. The toe modulus was obtained as the slope of the curves at 20% strain.

##### Porosity

Porosity was estimated for 4-layered scaffolds prior to compression testing. First, the scaffolds’ lengths (*l*), widths (*w*), and heights (*h*), were measured to calculate their volume assuming that these were fully solid rectangular prisms ($V_{solid}$), using Equation (1).

(1) $$V_{solid}=l\times w\times h\;$$

Then, the volume of the fully dense filaments that compose the scaffolds ($V_{scaffold}$) was calculated using Equation (2). This equation can be subdivided into two parts of the sum: the first part corresponds to the volume of the cylindrical filaments (where *r* and *x* correspond to the radius and linear segment of the scaffold’s filament, respectively) multiplied by the number of filaments per layer ($N_{FL}$) and the number of layers ($N_{L}$); the second part corresponds to the volume of the arched segments of the filaments, created by every directional and interlayer change (where *R* corresponds to half the distance between the center of two parallel filaments), multiplied by the number of directional changes per layer ($N_{DCL}$), the number of interlayer changes ($N_{ILC}$), and number of layers.

(2) $$V_{Scaffold}=\pi r^{2}x\times N_{FL}\times N_{L}+\left(\frac{2\pi r^{2}\times Rr^{2}\;}{2}\times N_{DCL}\times N_{ILC}\times N_{L}\right)\;$$

Once both volumes were calculated, the porosity (*P*) was estimated using Equation (3).

(3) $$P=\left(1-\frac{V_{scaffold}}{V_{solid}}\;\right)\times 100$$

##### Swelling Ratio and Scaffold Erosion

Swelling and erosion of the samples was also evaluated, having the freeze-dried scaffolds been divided into quarters, each with an average weight of 200 mg. For swelling measurements, scaffolds of each composition were submerged in 20 mL of a phosphate-buffered saline (PBS; pH 7.4) solution, at 37 °C, for periods of 5, 10, 20, 30 min and for 1, 2, 3, 4, 6, and 8 h, having 3 replicas been used for each time point. All samples were weighed before and after being submerged for each respective time frame. The swelling ratio was calculated according to Equation (4), where $w_{d}$ and $w_{h}$ corresponds to the scaffolds’ dry and hydrated weight, respectively.

(4) $$SR=\frac{w_{h}-w_{d}}{w_{d}}\;$$

Scaffold erosion was evaluated under a PBS media at 37 °C. For this, scaffolds of each composition were submerged in 20 mL of PBS solution for periods of 1, 2, 3, 5, 7, 14 and 28 days. In this experiment, 6 replicas per composition were used, having weighed each of them at the designated times and then resubmerging the same samples in PBS. Erosion was calculated according to Equation (5), where $w_{1}$ corresponds to the scaffold’s weight after 1 day of soaking in PBS and $w_{x}$ to the scaffold’s hydrate weight after *x* days of soaking (where *x* = 1, 2, 3, 5, 7, 14 and 28).

(5) $$ER=\left(\frac{w_{x}}{w_{1}}\right)\times 100\;$$

#### 3.2.6. In Vitro Studies

##### Cell Viability Assays

To evaluate the biocompatibility of the scaffolds containing pSPIONs, cell viability was studied using resazurin assays which allowed for the structures’ cytotoxicity effect to be assessed on a Saos-2 (primary osteogenic sarcoma) cell line using the extract dilution exposure method [51]. Initially, scaffolds were sterilized using a 70% ethanol solution and left to dry for 3 days. Then, at a concentration of 100 mg/mL, the sterilized scaffolds were submerged in a McCoy 5A medium, which was supplemented with 10% fetal bovine serum and 1% Penicillin-Streptomycin (10,000 mL^−1^), for 48 h at 37 °C in 5% CO_2_, thereby producing the extracts of each scaffold. The cells, seeded in 96-well plates at a density of 9000 cells/well, were grown using fresh medium and left in a humidified incubator at 37 °C with 5% ${\mathrm{CO}}_{2}$ for 24 h. In the next day, the cells were exposed to the extracts following a dilution factor of 0, 2, 4, 6, and 8, and the plates were incubated for 48 h. After this period, the medium was removed and resazurin was added to each well. After 3 h of incubation, the absorbance was measured at 571 and 601 nm, using a Biotek ELX 800 UV plate reader (Winooski, VT, USA). Cell viability was calculated the percentage of the negative control, given by Equation (6).

(6) $$\mathrm{Cell}\;\mathrm{Viability}\;\left(\%\right)=\frac{{\mathrm{OD}}_{570-600}\;\mathrm{sample}}{{\mathrm{OD}}_{570-600}\;\mathrm{negative}\;\mathrm{control}}\times 100\;$$

##### Cell Adhesion and Proliferation

To study the cell adhesion ratio and proliferation rate, scaffolds were first sterilized following the same procedure described for cell viability assays. Following this, scaffolds were left to swell in McCoy 5A medium for 24 h and then split into eight parts. Five replicas of each scaffold composition were seeded using Saos-2 cell line, at a density of 50,000 cells/well in a 48 well plate and left in a humidified incubator at 37 °C with 5% ${\mathrm{CO}}_{2}$ for 24 h. The following day, cell adhesion was assessed using resazurin assays, as described for cell viability assays. The adhesion ratio was calculated as the average of the quotient between the absorbance in each experimental well (EW) and the difference between the absorbance of cell control wells (CC) and the absorbance of the cells that sedimented to the bottom of the well upon cell seeding the scaffolds (CBW), as given by Equation (7):

(7) $$\mathrm{Adhesion}\;\mathrm{ratio}\;\left(\%\right)=Av\left(\frac{OD_{570-600\;\mathrm{EW}}}{OD_{570-600\;\mathrm{CC}}-OD_{570-600\;\mathrm{CBW}}}\right)\times 100$$

For the proliferation testing, these same cultures underwent resazurin assays after 4, 7, 11, and 14 days of culture time. In this study, cell adhesion and proliferation were repeated five times to ensure a good statistical and biological analysis, having replicated each different composition twenty times. The relative cell proliferation was calculated as the average of the sum of the absorbance in each experimental well, normalized to the average of the sum of the absorbance of cell control wells, using day 1 cell population data. This is represented in Equation (8) where n represents the number of wells and d corresponds to the days in which a resazurin assay was performed (*d* = 4, 7, 11, and 14).

(8) $$\mathrm{Relative}\;\mathrm{cell}\;\mathrm{population}=\frac{1}{n}\overset{n}{\underset{i=1}{\sum }}\;\overset{d}{\underset{j=1}{\sum }}\frac{{\left(OD_{570-600\;\mathrm{EW}}\right)}_{i,j}}{{\left(OD_{570-600\;\mathrm{CC}}\right)}_{i,1}}$$

##### Alkaline Phosphatase Assay

Alkaline phosphatase (ALP) assays were performed by transferring 100 µL of McCoy 5A medium, that was in contact with each seeded scaffold for 24 h prior to testing, to a 96-well plate. The absorbance was measured at 405 nm using a Biotek ELX 800 UV plate reader (Winooski, VT, USA). Following the measurement, 100 µL of a 1 mg/mL solution of p-NPP in TRIS-HCL (pH = 8.74) was added to each well and the plate was left in a humidified incubator at 37 °C with 5% ${\mathrm{CO}}_{2}$. The absorbance was measured at 405 nm after 30 min of incubation. This procedure was performed following each cell population evaluation, at 5, 8, 12, and 15 days after culture initiation and using 2 replicas per well. ALP expression is calculated as the variation in medium absorbance at 405 nm minus the same variation obtained for no cell controls. Relative ALP expression was calculated as the ratio between the ALP expression and cell population on the previous day, as given by Equation (9).

(9) $$\mathrm{Relative}\;\mathrm{ALP}\;\mathrm{expression}=\frac{\mathrm{ALP}\;\mathrm{expression}}{\mathrm{Cell}\;\mathrm{population}\;\mathrm{on}\;\mathrm{the}\;\mathrm{previous}\;\mathrm{day}}$$

##### Immunofluorescence Study

Cell morphology was examined by immunofluorescence of phalloidin-labelled actin filaments of the cytoskeleton and Helix NP^TM^ Green counterstained DNA. For fluorescence microscopy, cells were fixed with 3.7% paraformaldehyde (PFA) in PBS for 20 min at room temperature, and subsequently washed three times with PBS to remove the remaining PFA. Following this, cells underwent membrane permeabilization by exposure to a 0.2% TritonX-100 solution in PBS for 10 min. Actin labelling was performed by submerging cells in phalloidin conjugate (Phalloidin CruzFluor™ 488 Conjugate) diluted in PBS using a ratio of 7:100 for 30 min. Finally, the DNA was counterstained using 5 nM of Helix NP^TM^ Green in PBS for 10 min and observed in a NIKON Eclipse TI-S inverted microscope (NIKON, Tokyo, Japan). Regarding the cell control, the DNA counterstaining was performed with DAPI in PBS using a ratio of 1:30 for 5 min.

#### 3.2.7. Statistical Analysis

All data related to in vitro evaluation were statistically analyzed using the software GraphPad Prism 9 (version 9.4.0, GraphPad Software, San Diego, CA, USA.) and presented using mean ± SD. Adhesion rate results were analyzed using a one-way analysis of variance (One-way ANOVA) with Tukey’s multiple comparison test and *p* < 0.05 was accepted as significant. Proliferation and ALP expression results were analyzed using a two-way analysis of variance (Two-way ANOVA) with Dunnett’s multiple comparison test and *p* < 0.05 was accepted as significant.

## 4. Conclusions

The present study aimed at developing a 3D printed composite scaffold with bone regenerative capabilities and magnetic properties with potential for cancer therapy. These structures were obtained by 3D printing a polymeric blend composed of CS and PVA with integrated HA particles and pSPIONs, and subsequent crosslinking through a combination of NaOH (CS), GLY (between CS and PVA), and F/T cycles (PVA). These scaffolds, with excellent stability under a saline medium, exhibited a mechanical behavior similar to that of the organic phase of bones. Furthermore, compression testing also demonstrated that pSPIONs incorporation did not seem to have a significant impact on the scaffolds’ mechanical properties.

FTIR data confirmed that the incorporation of the pSPIONs promoted electrostatic interactions between the pSPIONs and CS present in the scaffolds’ matrix. This interaction promoted to a decrease in the swelling capacity of the 3D printed scaffolds with the increase of their concentration. Furthermore, due to that same interaction, the incorporation of the pSPIONs into the 3D printed CS/PVA/HA scaffolds acted as a mechanical reinforcement.

The magnetic studies showed that scaffolds containing pSPIONs displayed a temperature increase under application of an AMF, dependent on NPs concentration, thus demonstrating potential for MHT applications.

All samples displayed no cytotoxicity for concentrations up to 50 mg/mL. Additionally, the incorporation of pSPIONs significantly improved Saos-2 cell adhesion and proliferation when compared to controls. Moreover, scaffolds P1_pS3 and P1_pS5 exhibited proliferation ratios equivalent to the control during the first 4 days of cell culture. Besides, all cells grown on pSPIONs-containing scaffolds expressed ALP, which indicates that bone cells grown on these structures may be capable of forming an apatite layer that is essential for bone regeneration.

Overall, the structure CS/PVA/HA/pSPIONs scaffolds showed promising results for applications in bone cancer therapy and regeneration, through a synergy between magnetic hyperthermia therapy and osteogenic activity enhancement.

## 5. Future Perspectives

Future research could focus on the induction of filament mesoporosity or coating the scaffolds’ surface with collagen or fibronectin, which could lead to a more suitable microenvironment for cell adhesion and proliferation through improved cell-surface interactions; and the incorporation of an anticancer drug, such as DOX for a thermally controlled localized drug delivery, thus complementing the scaffolds’ therapeutic capabilities.

## Figures and Tables

**Table 1:** Blend at two different ratios of the chitosan solution with three different PVA/HA pastes to attain six paste formulations with varying PVA content and the CS/PVA/HA pastes solid content.

Paste Designation	Paste Formulation (wt.%)				Paste Solids Content (wt.%)		
	CS ^[a]^	PVA/HA		CS:PVA/HA Ratio	CS	PVA	HA
		PVA ^[a]^	HA ^[b]^				
P1	6	12.5	15	3:1	49.83	37.13	13.04
P2	6	15.0	15	3:1	45.28	41.68	13.04
P3	6	17.5	15	3:1	41.28	45.68	13.04
P4	6	12.5	15	3:2	34.89	52.07	13.04
P5	6	15.0	15	3:2	30.58	56.38	13.04
P6	6	17.5	15	3:2	27.04	59.92	13.04

[a] In relation to the initial solution concentration. [b] In relation to the initial solution concentration.

**Table 2:** Solid content of the CS/PVA/HA/pSPIONs pastes produced by adding pSPIONs to paste P1 at three different weight percentages (15, 30, and 45 wt.%) relative to the total amount of HA in the paste.

Samples Designation	Samples	CS (wt.%)	PVA (wt.%)	HA (wt.%)	pSPIONs (wt.%)
P1_B	CS/PVA/HA	49.83	37.13	13.04	-
P1_pS1	CS/PVA/HA/15%pSPIONs	48.87	36.42	12.79	1.92
P1_pS3	CS/PVA/HA/30%pSPIONs	47.95	35.73	12.55	3.77
P1_pS5	CS/PVA/HA/45%pSPIONs	47.07	35.07	12.32	5.54

**Table 3:** Average filament diameter for each of the four different 3D printed scaffold compositions.

Samples	18G Needle	P1_B	P1_pS1	P1_pS3	P1_pS5
Filament Diameter (µm)	840 ± 2	918 ± 37	839 ± 17	832 ± 32	876 ± 8

**Table 4:** Porosity and toe modulus of the tested scaffolds.

Samples	Estimated Porosity ^[a]^ (%)	Toe Modulus (kPa)	pSPIONs Content (wt. %)
P1_B	70 ± 3	27 ± 8	0.00
P1_pS5	73 ± 2	92 ± 4	5.54

[a] Average porosity measured for 4-layered scaffolds.

**Table 5:** Adhesion ratio of Saos-2 cells to the scaffolds: P1_B, P1_pS1, P1_pS3, and P1_pS5; and results of the statistical significance tests regarding the differences between the adhesion ratios. Adhesion ratio was calculated using day 1 cell population data. Uncertainty is the experimental standard deviation of the mean.

Samples	Adhesion Ratio (%)	P1_B	P1_pS1	P1_pS3	P1_pS5
P1_B	63 ± 12	-	*	**	**
P1_pS1	77 ± 11	*	-	ns	ns
P1_pS3	85 ± 8	**	ns	-	ns
P1_pS5	86 ± 11	**	ns	ns	-

ns—non significant; *—*p* < 0.05; **—*p* < 0.001.

**Table 6:** Relative cell proliferation calculated as the average of the absorbance in each experimental well, normalized to the average of the absorbance of cell control wells, using day 1 cell population data. Cell proliferation ratios during the initial days of culture calculated as the ratio between cell population on day 4 and on day 1 (PR 1–4) and on days 7 and 1 (PR 1–7). Proliferation rate uncertainty is the combined standard uncertainty.

Samples	Day 1	Day 4	Day 7	PR 1–4	PR 1–7
P1_B	0.39 ± 0.09	0.46 ± 0.13	0.43 ± 0.10	1.21 ± 0.10	1.11 ± 0.06
P1_pS1	0.40 ± 0.11	0.56 ± 0.13	0.58 ± 0.09	1.51 ± 0.13	1.23 ± 0.08
P1_pS3	0.38 ± 0.08	0.67 ± 0.15	0.74 ± 0.12	1.96 ± 0.08	2.10 ± 0.14
P1_pS5	0.37 ± 0.10	0.65 ± 0.14	0.72 ± 0.14	1.97 ± 0.20	1.87 ± 0.20
Cell Control	1.000 ± 0.004	1.87 ± 0.19	2.42 ± 0.28	1.87 ± 0.19	2.42 ± 0.28

**Table 7:** Results of the statistical significance tests regarding the differences between the relative ALP expressed by Saos-2 cells on scaffolds: P1_B, P1_pS1, P1_pS3, and P1_pS5. Statistical analysis was performed by comparing the relative ALP expression of the Saos-2 cells for the whole duration of the cell culture.

Samples	P1_B	P1_pS1	P1_pS3	P1_pS5
P1_B	-	****	****	****
P1_pS1	****	-	ns	ns
P1_pS3	****	ns	-	ns
P1_pS5	****	ns	ns	-

ns—non significant; ****—*p* < 0.0001.

## Data Availability

Not applicable.
